# LRCH1 polymorphisms linked to delayed encephalopathy after acute carbon monoxide poisoning identified by GWAS analysis followed by Sequenom MassARRAY® validation

**DOI:** 10.1186/s12881-019-0931-7

**Published:** 2019-12-16

**Authors:** Jiapeng Gu, Jiao Zeng, Xi Wang, Xin Gu, Xiaoli Zhang, Ping Zhang, Fan Zhang, Yongkai Han, Yazhou Han, Hongxing Zhang, Wenqiang Li, Renjun Gu

**Affiliations:** 1 Henan Mental Hospital, The Second Affiliated Hospital of Xinxiang Medical University, No. 388 Jianshe Middle Road, Muye District, Xinxiang City, 453002 Henan Province China; 2 Qinyang People’s Hospital, Jiaozuo City, 454550 Henan Province China; 3 The Psychology College of Xinxiang Medical University, Xinxiang City, 453002 Henan Province China; 4 International Joint Research Laboratory for Psychiatry and Neuroscience of Henan, Henan Key Lab of Biological Psychiatry of Xinxiang Medical University, Xinxiang City, 453002 Henan Province China

**Keywords:** DEACMP, ACMP, LRCH1 polymorphisms, SNP genotyping, Genetic model

## Abstract

**Background:**

We explored the association of leucine-rich repeats and calponin homology domain containing 1 (LRCH1) gene polymorphisms with genetic susceptibility to delayed encephalopathy after acute carbon monoxide poisoning (DEACMP), which might provide a theoretical basis for the pathogenesis, diagnosis, and prognosis research of DEACMP.

**Methods:**

Four single nucleotide polymorphisms, rs1539177 (G/A), rs17068697 (G/A), rs9534475 (A/C), and rs2236592 (T/C), of LRCH1, selected as candidate genes through genome-wide association analysis, were genotyped in 661 patients (DEACMP group: 235 cases; ACMP group: 426 cases) using Sequenom Massarray®. The association analysis of four SNPs and LRCH1 was performed under different genetic models.

**Results:**

LRCH1 polymorphisms (rs1539177, rs17068697, rs9534475) under additive and dominant genetic models were significantly associated with an increased risk of DEACMP, but no significant association under allele and recessive models was found. The LRCH1 rs2236592 polymorphism was susceptible to DEACMP only under the dominant model (TT/TC + CC, OR = 1.616, 95% CI: 1.092–2.390, *P* = 0.015784). In addition, the A allele gene of rs9534475 polymorphism in LRCH1 might increase the risk for DEACMP (OR = 1.273, 95% CI: 1.013–1.601, *P* = 0.038445).

**Conclusions:**

We found a significant association between the four LRCH1 polymorphisms and DEACMP. The allelic A of rs9534475 polymorphism in LRCH1 might be a risk factor for DEACMP.

## Background

Carbon monoxide (CO) poisoning occurs after prolonged inhalation of CO at a lethal dosage of 100 ppm or higher [1]. CO poisoning, as a leading gas poisoning event in the United States, affects 50,000 people per year [2]. Reportedly, CO poisoning events are more common in China than in western countries due to the large Chinese population base and usage of primary energy sources [3]. Delayed encephalopathy after acute carbon monoxide poisoning (DEACMP) is one of most severe complications of CO poisoning and occurs after a 2–60 day latency phase, in which the symptoms of acute CO poisoning transiently disappear [4]. The main clinical manifestations of DEACMP are mental disturbances, slow responses, cognitive dysfunction, gatism, dystonia, and even dementia [5]. So far, there is no specific therapy for DEACMP treatment; it has a poor prognosis with different degrees of intellectual disability and cognitive disorder as well as long-term agrypnocoma after treatment [6].

The specific pathogenesis of DEACMP has not yet been clarified. Generally recognized theories include the ischemia and hypoxia theory, the inflammation and immune injury theory, and the excitatory neurotransmitter theory [7]. Recently, accumulating evidence suggests that DEACMP is associated with a genetic factor. Populations with specific gene polymorphisms, such as LP-PLA2 rs1805017/ rs1051931, LRP1B rs1541976, and NRXN3 rs11845632/rs2196447, have genetic susceptibility to DEACMP [8–10]. To identify other related gene polymorphisms, a genome-wide association study (GWAS) was conducted in our previous study, which involved DNA samples from 175 DEACMP and 277 ACMP patients; allele and genotype frequencies of all detected single nucleotide polymorphisms (SNPs) were compared between those two groups [8]. Subsequently, four SNPs (rs1539177, rs17068697, rs9534475, and rs2236592) in the Leucine-rich repeats and calponin homology domain containing 1 (LRCH1) gene were identified as potential candidate sites associated with DEACMP (Additional file 1: Table S1).

LRCH1 belongs to a member of the LRCH family, which has a leucine-rich repeat domain and a calponin homology domain [11]. Little is currently known regarding the healthy biological function of this protein. Multiple SNP sites of LRCH1 are connected with osteoarthritis (OA) [12]. The pathogenesis of both OA and DEACMP is involved in immunization; hence, we speculate that LRCH1 may participate in the occurrence of DEACMP through an inflammatory immune mechanism. The latest research on LRCH1 by Xu and colleagues has provided more theoretical references for our conjecture [13]. They observed that LRCH1 acted as a novel DOCK8-interacting protein to inhibit T cell migration. T cells are closely related to the occurrence and development of various inflammatory diseases [14]. However, the association analysis between LRCH1 and DEACMP has not yet been reported. Collectively, we examined whether several LRCH1 SNPs influence the nosogenesis of DEACMP.

In recent years, the Sequenom Massarray® has proven to be an efficient method for genetic susceptibility gene detection screened by SNP genotyping [15]. Thus, in this study, the Sequenom method was used to further verify four candidate LRCH1 gene polymorphisms in DEACMP and ACMP patients with large samples. This study explored the associations of LRCH1 gene polymorphisms with genetic susceptibility to DEACMP, which might provide a theoretical basis for the pathogenesis, diagnosis, and prognosis research related to DEACMP.

## Methods

### Subjects

The study was approved by the Ethical Committee of the Second Affiliated Hospital of Xinxiang Medical University. The diagnostic criteria of DEACMP were as follows: (1) patients who had a history of coma due to acute CO poisoning within the last 2 months; (2) patients who experienced a latent phase (2–60 days) before the appearance of delayed symptoms; (3) delayed acute dementia of the central nervous system (CNS) damage as the primary clinical manifestation; and (4) EEG, CT, or MRI abnormalities. Meanwhile, the diagnostic criteria of “Occupational Acute Carbon Monoxide Poisoning” (GBZ23–2002) was used to diagnose ACMP disease. The ACMP patients experienced a history of coma due to acute CO poisoning, and no DEACMP event occurred following recovery for more than 90 days.

In addition to meeting the above diagnostic criteria, the subjects in the DEACMP and ACMP groups were both of Han Chinese descent from North Henan province and older than 40 years of age. Patients with other diagnosed nervous system dysfunctions, a history of infection within the previous 15 days, or recently administered hormone or immunosuppressive therapy were excluded.

In total, 661 patients (DEACMP group: 235 cases; ACMP group: 426 cases) were recruited from several hospitals in three cities during December 2017 to November 2006, including the First Affiliated Hospital of Xinxiang Medical University, the Second Affiliated Hospital of Xinxiang Medical University, the Third Affiliated Hospital of Xinxiang Medical University, Xinxiang Central Hospital, Xinxiang First People’s Hospital, Xinxiang Second People’s Hospital, the 371 Central Hospital of People’s Liberation Army, Wuzhi County People’s Hospital, Henan Grace Hospital, the People’s Hospital of Anyang City, Qinyang People’s Hospital, Jiaozuo Municipal Second Peoples’ Hospital, and General Hospital of Ansteel Group. All participants provided written informed consent.

The mean age, gender, and education levels of patients in the DEACMP and ACMP groups were matched. The peripheral blood samples from DEACMP patients were collected into anticoagulant vacuum tubes, which were collected from 6:00 am to 8:00 am following an overnight fast. Using the same method, the blood samples from ACMP patients were collected within 24 h of fully conscious recovery. All blood samples were labeled and stored at − 80 °C.

### Individual genotyping

Genomic DNA from peripheral blood samples of each patient was extracted using a TIANamp Blood DNA Midi Kit (DP332, Tiangen Biotech, Beijing, China). The method for SNP genotyping of all samples was based on the Sequenom Massarray® platform [16].

The design of all amplification primers of 4 SNPs of the LRCH1 gene was followed by gene sequencing using the dbSNP database and added to the 5′ 10-mer tag (ACGTTGGATG), as shown in Table 1. The locus-specific PCR was amplified through multiple PCR amplification. The initial multiple PCR amplification was conducted using a Sequenom amplification kit. The cycling conditions were set as follows: beginning denaturation at 94 °C for 4 min; 45 cycles of 94 °C for 20 s, 56 °C for 30 s, and 72 °C for 60 s, followed by 72 °C for 5 min.

**Table 1:** Primers for LRCH1 polymorphisms (rs1539177, rs17068697, rs9534475 and rs2236592)

LRCH1 SNP	PCR amplification primer sequences(5′-3′)	Single base extension primer sequence
LRCH1 (rs1539177)	ACGTTGGATGCTCTTTCAGGCTCTAATTT	cAGACATAAATGATCAATATGACCC
	ACGTTGGATGTGCCCCTCAAGGAGTGATT	cAGACATAAATGATCAATATGACCT
LRCH1 (rs17068697)	ACGTTGGATGGGGTCCATGCTTATCTCTTC	GGCGTCTGCTAAGTAAATTGCGTATA
	ACGTTGGATGCTGCACCCAGCATAAATAAC	GGCGTCTGCTAAGTAAATTGCGTATG
LRCH1 (rs9534475)	ACGTTGGATGACTATAGTCACTTAGGCCCC	ctcGGCCCCATAGTCAGAC
	ACGTTGGATGCCAGTGCTTAGACTCAATGC	ctcGGCCCCATAGTCAGAA
LRCH1 (rs2236592)	ACGTTGGATGCGACTTGAGAGTTAATGGAG	AGAAAGAGGATGATTTAAGGTACA
	ACGTTGGATGTGACTCCTCTCTGAGATTCC	AGAAAGAGGATGATTTAAGGTACG

*SNP* Single nucleotide polymorphism, *PCR* Polymerase chain reaction

Subsequently, SAP (shrimp alkaline phosphatase) treatment was performed to remove free dNTPs in the reaction system. In total, 2 μl of SAP Mix containing 1.53 μl water, 0.17 μl SAP buffer (10x), and 0.3 μl SAP enzyme (1.7 U/μl) was added into the above PCR system and incubated at 37 °C for 40 min, followed by 85 °C for 5 min. After the SAP treatment, 2 μl EXTEND mix, including 0.619 μl water, 0.94 μl Extend primer mix 1, 0.2 μl iPLEX Buffer Plus, 0.2 μl iPLEX terminator, and 0.041 μl iPLEX enzyme, was added to conduct the single base extension reaction. The PCR procedure was set as 94 °C for 30 s; 39 cycles of 94 °C for 5 min (step 1), 52 °C for 5 s, and 80 °C for 5 s (steps two and three were repeated four times for one cycle); and 72 °C for 3 min. Afterward, resin purification was performed.

Finally, the PCR product was transferred into a Spectro CHIP (Sequenom) using a MassARRAY Nanodispenser RS1000 microarrayer (Capital Bio-Technology, Beijing, China). The genotypes and alleles were detected by MALDI-TOF mass spectrometry, and the results were analyzed using Sequenom Typer 4.0 software. With the analyzed results, the four SNPs were individually genotyped in all samples.

### Statistical analysis

SPSS 20.0 software (SPSS, Inc., Chicago, IL, USA) was used to analyze the original data, and the Chi-square (χ2) goodness of fit test combined with the χ^2^ test was used to examine whether the distributions of genotypes in both groups conformed to the Hardy–Weinberg equilibrium law. The Karl Pearson χ^2^ test of the 2 × 2 tables and 2 × 3 contingency table with one degree of freedom was applied to assess the relationship between genetic model or allele gene and the DEACMP risk. Odds ratios (ORs) and corresponding 95% confidence intervals (95% CIs) were calculated for the four SNPs.

## Results

### Clinical characteristics

The four candidate SNPs (rs1539177, rs17068697, rs9534475, and rs2236592) of the LRCH1 gene were located in the intron region of chromosome 13 (Table 2). The demographic and clinical data regarding numbers, age, gender distribution, and educational level of patients in two groups are shown in Table 3. Among the 661 participants, a total of 658 (DEACMP: 232; ACMP: 426), 656 (DEACMP: 234; ACMP: 422), 661 (DEACMP: 235; ACMP: 426), and 659 (DEACMP: 235; ACMP: 424) patients were genotyped with rs1539177 (G/A), rs17068697 (G/A), rs9534475 (A/C), and rs2236592 (T/C) polymorphisms, respectively. In addition, there was no statistical difference of mean age, gender distribution, and educational level between two groups for rs1539177 (mean age: *P* = 0.1299; gender distribution: *P* = 0.077; educational level: *P* = 0.121), rs17068697 (mean age: *P* = 0.1285; gender distribution: *P* = 0.073; educational level: *P* = 0.119), rs9534475 (mean age: *P* = 0.1285; gender distribution: *P* = 0.071; educational level: *P* = 0.123), and rs2236592 (mean age: *P* = 0.1271; gender distribution: *P* = 0.072; educational level: *P* = 0.129) (Table 3). Notably, the genotype distributions of these four SNPs between the two groups were within the Hardy–Weinberg equilibrium law (all *P* > 0.05, Table 4).

**Table 2:** Physical locations of LRCH1 polymorphisms

Variant	Chrom	Position	Annotation	MAF
rs1539177 G/A	13	46,725,920	intron	0.38 (A)
rs17068697 A/G	13	46,719,680	intron	0.26 (G)
rs9534475 A/C	13	46,718,476	intron	0.40 (C)
rs2236592 T/C	13	46,721,781	intron	0.44 (C)

*MAF* Minor allele frequency

**Table 3:** Demographic variables of DEACMP and ACMP patients genotyped for the rs1539177, rs17068697, rs9534475, and rs2236592 polymorphisms

Characteristic	rs1539177				rs17068697				rs9534475				rs2236592			
	DEACMP (*n* = 232)	ACMP (*n* = 426)	Statistics	*P* value	DEACMP (*n* = 234)	ACMP (*n* = 422)	Statistics	*P* value	DEACMP (*n* = 235)	ACMP (*n* = 422)	Statistics	*P* value	DEACMP (*n* = 235)	ACMP (*n* = 424)	Statistics	*P* value
Age	62.24 ± 10.35	65.18 ± 6.48	t = 1.502	0.1299	62.32 ± 10.27	65.23 ± 6.52	*t* = 1.487	0.1285	62.32 ± 10.27	65.23 ± 6.52	t = 1.4994	0.1285	62.35 ± 10.51	65.29 ± 6.31	*t* = 1.472	0.1271
Total	232	426			234	422			235	426			235	424		
Male	132	212	*χ2* = 3.062	0.077	134	211	*χ2* = 3.187	0.073	134	212	*χ2* = 3.197	0.071	134	211	*χ2* = 3.192	0.072
Female	100	214			100	211			101	214			101	213		
Educational level																
Uneducated	69	149	*χ2* = 4.448	0.121	71	147	*χ2* = 4.867	0.119	71	147	*χ2* = 4.356	0.123	71	147	*χ2* = 4.162	0.129
Primary school	85	166			83	165			83	165			85	166		
Middle school	78	111			80	110			80	110			79	111

**Table 4:** Results of Hardy-Weinberg Equilibrium test for genotypes distributions of LRCH1 polymorphisms

SNP	Genotypes	risk allele	Risk allele frequency ACMP/DEACMP	Actual value	Test value
rs1539177	GG	A		162	χ2 = 0.344340179 *P* = 0.557334638
	AG		0.297/0.433	206	
	AA			58	
rs17068697	GG	A		95	χ2 = 2.145715207 *P* = 0.142968447
	AG		0.5071/0.562	226	
	AA			101	
rs9534475	AA	C		167	χ2 = 0.196790791 *P* = 0.657323794
	AC		0.378/0.4362	196	
	CC			63	
rs2236592	TT	C		115	χ2 = 1.62565207 *P* = 0.202305581
	TC		0.4646/0.5149	224	
	CC			85	

### Association analysis between rs1539177, rs17068697, rs9534475, and rs2236592 polymorphisms and DEACMP

The results of the association analysis between four SNPs under different genetic models and increased risk of DEACMP are presented in Table 5. The allele frequencies of rs1539177, rs17068697, and rs2236592 polymorphisms between patients in both groups were similar without significant differences (all *P* > 0.05). Notably, there was no correlation between the rs1539177, rs17068697, rs9534475, and rs2236592 polymorphisms under the recessive model and increased risk of DEACMP (*P* = 0.50527, *P* = 0.466963, *P* = 0.742672, and *P* = 0.61508, respectively).

**Table 5:** Correlation analysis of LRCH1 polymorphisms under different genetic models and DEACMP risk

SNPs	Genetic models		DEACMP	ACMP	*P*_obs_	OR(95%CI)
rs1539177	allele	(G/A)	263/201	530/322	0.050346	0.795 (0.632–1.001)
	additive	(GG/AG/AA)	67/129/36	162/206/58	0.043729	1.279 (1.007–1.624)
	dominant	(GG/AG + AA)	67/165	162/264	0.018579	1.511 (1.070–2.133)
	recessive	(GG + AG/AA)	196/36	368/58	0.50527	1.165 (0.743–1.828)
rs17068697	allele	(G/A)	205/263	416/428	0.056602	0.802 (0.639–1.006)
	additive	(GG/AG/AA)	33/139/62	95/226/101	0.043125	1.285 (1.008–1.6381)
	dominant	(GG/AG + AA)	33/201	95/327	0.009228	1.770 (1.147–2.729)
	recessive	(GG + AG/AA)	172/62	321/101	0.466963	1.146 (0.794–1.653)
rs9534475	allele	(A/C)	265/205	530/322	0.038445	1.273 (1.013–1.601)
	additive	(AA/AC/CC)	67/137/37	167/196/63	0.035878	1.285 (1.017–1.623)
	dominant	(AA/AC + CC)	67/168	167/259	0.005935	1.617 (1.147–2.280)
	recessive	(AA+AC/CC)	198/37	363/63	0.742672	1.077 (0.693–1.674)
rs2236592	allele	(T/C)	228/242	454/394	0.080373	1.223 (0.976–1.533)
	additive	(TT/TC/CC)	44/140/51	115/224/85	0.064827	1.254 (0.986–1.597)
	dominant	(TT/TC + CC)	44/191	115/309	0.015784	1.616 (1.092–2.390)
	recessive	(TT + TC/CC)	184/51	339/85	0.61508	1.105 (0.748–1.634)

The association analysis between rs17068697 and DEACMP showed that rs17068697 was associated with susceptibility to DEACMP under the additive model (GG/AG/AA, OR = 1.279, 95% CI: 1.007–1.624, *P* = 0.043729) and the dominant model (GG/AG + AA, OR = 1.511, 95% CI: 1.070–2.133, *P* = 0.018579). Similarly, the rs17068697 was associated with susceptibility to DEACMP under the additive model (GG/AG/AA, OR = 1.285, 95% CI: 1.008–1.6381, *P* = 0.043125) and the dominant model (GG/AG + AA, OR = 1.770, 95% CI: 1.147–2.729, *P* = 0.009228).

The A allele frequency of rs9534475 polymorphism was significantly higher than the C allele frequency in DEACMP patients (OR = 1.273, 95% CI: 1.013–1.601, *P* = 0.038445). In addition, the rs9534475 polymorphism under the additive model (AA/AC/CC, OR = 1.285, 95% CI: 1.017–1.623, *P* = 0.035878) and dominant model (AA/AC + CC, OR = 1.617, 95% CI: 1.147–2.280, *P* = 0.005935) were associated with an increased risk of DEACMP. Analysis of rs2236592 demonstrated that only rs2236592 polymorphism under the dominant model (TT/TC + CC, OR = 1.616, 95% CI: 1.092–2.390, *P* = 0.015784) increased susceptibility to DEACMP.

## Discussion

We performed the genotype frequency analyses for the four LRCH1 polymorphisms (rs1539177, rs17068697, rs9534475, and rs2236592) in two groups and the association analysis of these four LRCH1 polymorphisms with the occurrence of DEACMP under different genetic models. The results revealed that the three LRCH1 polymorphisms (rs1539177, rs17068697, rs9534475) under additive and dominant genetic models were associated with an increased risk for DEACMP. The LRCH1 rs2236592 polymorphism was susceptible to DEACMP under the dominant model (TT/TC + CC). In addition, the A allele gene of rs9534475 polymorphism might increase the risk of DEACMP.

Although the pathogenesis of DEACMP is complex and poorly revealed, mechanisms of immune injury-induced inflammatory reactions can cause the neurological damage associated with DEACMP [17, 18]. Thom et al. have reported that DEACMP is immune-mediated and associated with chemically modified myelin basic protein (MBP) [19]. The degraded MBP becomes an autoantigen, which acts on macrophages and CD-4 lymphocytes to infiltrate the brain. Similarly, Xiang et al. have suggested that DEACMP is caused by MBP-level changes from acute CO intoxication with induced neutrophils and inflammatory response activation [20]. In addition, the elevated levels of IL-2, IL-4, IL-1β, IL-6, IL-10, IL-8, and TNF-α in the cerebrospinal fluid of DEACMP patients are reported to be predictive markers of DEACMP [21–23]. Those studies have demonstrated that immune molecules mediated immunopathological damage plays a crucial role in the occurrence of DEACMP.

In our previous GWAS analysis, LRCH1 was identified as a candidate gene related to DEACMP [8], and in this study, the results of SNP genotyping revealed that four LRCH1 polymorphisms (rs1539177, rs17068697, rs9534475, and rs2236592) were closely related to LRCH1. LRCH1 is located in chromosome 13q14 and extensively expressed in vivo; however, the specific functions of its encoded proteins are still poorly understood. So far, increased studies have reported that LRCH1 gene polymorphisms are associated with osteoarthritis [24, 25]. Spector et al. reported that an rs912428 polymorphism (C/T) in intron 1 of LRCH1 was linked with an increased risk of knee osteoarthritis via a possible structural role [24]. Additionally, Panoutsopoulou et al. found that another rs754106 polymorphism in intron 1 of LRCH1 was susceptible to osteoarthritis and the T allele gene increases risk of knee osteoarthritis by 1.14 times when compared with the C allele gene [25]. It is widely known that dysregulated innate immune responses can result in osteoarthritis via mediation of chronic inflammation [26]. In addition, the immunologic changes of high levels of some cytokines (IL1, IL-10, TNF-α, and IL-17) and high CD^4+^/CD8^+^ T cell ratio are detected in arthritis and osteoarthritis samples [27]. Considering that the pathogenesis of osteoarthritis and DEACMP are both involved in immunologic mechanisms, these findings imply that LRCH1 might also be associated with the occurrence of DEACMP through its participation in immunologic mechanisms.

Recently, LRCH1 is found to compete with Cdc42 for DOCK8 binding, which induces T cell migration and relieves autoimmune encephalomyelitis [13]. Similarly, CD4^+^ CD25^+^ Treg and CD8^+^ CD28^−^Treg infiltration into the CNS has been reported to play a crucial role in the pathogenesis of autoimmune encephalomyelitis via the regulation of local inflammation [28]. Notably, DEACMP is also a dysregulated CNS disease with autoimmune responses, which involve many of the immunocytes and cytokines mentioned above. Thus, these findings further supported our perspective that LRCH1 might also be associated with the occurrence of DEACMP via the regulation of T cell migration and migration into the CNS to mediate inflammation and immune reactions.

The associations of four LRCH1 polymorphisms and DEACMP are newly discovered. Our results showed that LRCH1 polymorphisms (rs1539177, rs17068697, and rs9534475) were susceptible to DEACMP under additive and dominant genetic models, indicating the risk alleles in rs1539177 (A), rs17068697 (A), and rs9534475 (C) might increase the occurrence of DEACMP based on additive and dominant autosomal inheritance patterns. The risk allele C in rs1539177 might also increase the risk of DEACMP based on the dominant autosomal inheritance pattern.

## Conclusions

In summary, our results revealed a significant association between four LRCH1 polymorphisms (rs1539177, rs17068697, rs9534475, and rs2236592) and DEACMP patients. The allelic A of rs9534475 polymorphism might be a risk factor for developing DEACMP. However, the results were limited to a small part of the Han Chinese population from North Henan province and should be explored in other populations in the future.

## Supplementary information

**Additional file 1: Table S1.** SNPs associated with DEACMP in the pooling-based GWAS in both genders.

## Data Availability

All data generated and/or analyzed during the current study are available in these links (https://www.ncbi.nlm.nih.gov/snp/rs1539177, https://www.ncbi.nlm.nih.gov/snp/rs17068697, https://www.ncbi.nlm.nih.gov/snp/rs9534475, https://www.ncbi.nlm.nih.gov/snp/rs2236592).

## Abbreviations

95% CIs: 95% confidence intervals
CO: Carbon monoxide
DEACMP: Delayed encephalopathy after acute carbon monoxide poisoning
GWAS: Genome-wide analysis study
LRCH1: Leucine-rich repeats and calponin homology domain containing 1
MBP: Myelin basic protein
ORs: Odds ratios
SAP: Shrimp alkaline phosphatase
SNPs: Single nucleotide polymorphisms
