# Supplementary material for: LRCH1 polymorphisms linked to delayed encephalopathy after acute carbon monoxide poisoning identified by GWAS analysis followed by Sequenom MassARRAY® validation
Source: BMC Med Genet. 2019 Dec 16;20:197. doi: 10.1186/s12881-019-0931-7 (PMC6916040; doi:10.1186/s12881-019-0931-7)
Supplement: Supplementary file 1 — Additional file 1: Table S1. SNPs associated with DEACMP in the pooling-based GWAS in both genders. [file 12881_2019_931_MOESM1_ESM.docx]

Table S1. SNPs associated with DEACMP in the pooling-based GWA in both genders.

| SNP | Chromosome | Position | *p* value | | Gene |
| --- | --- | --- | --- | --- | --- |
|  |  |  | Male | Female |  |
| rs1368387 | 5 | 147876501 | -0.61727 | -0.62193 | 5-hydroxytryptamine (serotonin) receptor 4 |
| rs867522 | 5 | 147946439 | -0.5712 | -0.61503 | 5-hydroxytryptamine (serotonin) receptor 4 |
| rs270378 | 6 | 7707714 | -0.5023 | -0.50627 | bone morphogenetic protein 6 |
| rs270398 | 6 | 7710839 | -0.5307 | -0.54817 | bone morphogenetic protein 6 |
| rs17126880 | 1 | 64922104 | -0.5907 | -0.54827 | cache domain containing 1 |
| rs2513796 | 8 | 95211595 | -0.50727 | -0.52173 | cadherin 17, LI cadherin (liver-intestine) |
| rs6028103 | 20 | 59319547 | -0.51957 | -0.50213 | cadherin 4, type 1, R-cadherin (retinal) |
| rs3747869 | 10 | 73190638 | -0.53963 | -0.53347 | cadherin-related 23 |
| rs6445588 | 3 | 53605719 | -0.51903 | -0.51207 | calcium channel, voltage-dependent, L type, alpha 1D subunit |
| rs4295733 | 9 | 140098397 | -0.51993 | -0.5838 | calcium channel, voltage-dependent, N type, alpha 1B subunit |
| rs1962336 | 10 | 50063019 | 0.5381667 | 0.5442333 | chromosome 10 open reading frame 128 |
| rs2804931 | 10 | 50043527 | 0.6074 | 0.5560333 | chromosome 10 open reading frame 128 |
| rs2725180 | 10 | 50039401 | -0.53403 | -0.51587 | chromosome 10 open reading frame 128 |
| rs32262 | 5 | 111109396 | -0.5688 | -0.5806 | chromosome 5 open reading frame 13 |
| rs32333 | 5 | 111122362 | -0.6051 | -0.59437 | chromosome 5 open reading frame 13 |
| rs2058501 | 7 | 120467472 | 0.6294 | 0.610933 | chromosome 7 open reading frame 58 |
| rs6949989 | 7 | 120428967 | 0.621233 | 0.569133 | chromosome 7 open reading frame 58 |
| rs4940465 | 18 | 55324824 | 0.5223667 | 0.5078333 | collagen and calcium binding EGF domains 1 |
| rs585632 | 18 | 55285808 | 0.5558 | 0.5650667 | collagen and calcium binding EGF domains 1 |
| rs688519 | 18 | 55319648 | 0.5435667 | 0.5634667 | collagen and calcium binding EGF domains 1 |
| rs10247883 | 7 | 9075442 | 0.663167 | 0.551467 | colony stimulating factor 2 receptor, beta, low-affinity |
| rs2413436 | 22 | 35642507 | 0.8456 | 0.5105 | colony stimulating factor 3 receptor, beta, low-affinity |
| rs4821567 | 22 | 35646334 | 0.665933 | 0.608567 | colony stimulating factor 4 receptor, beta, low-affinity |
| rs11550299 | 11 | 66010661 | -0.61673 | -0.53583 | dipeptidyl-peptidase 3 |
| rs2305535 | 11 | 66028813 | -0.6083 | -0.5625 | dipeptidyl-peptidase 3 |
| rs799618 | 7 | 110272614 | 0.5941333 | 0.5254333 | IMP2 inner mitochondrial membrane peptidase-like |
| rs12705744 | 7 | 110307026 | 0.5259 | 0.5017667 | IMP2 inner mitochondrial membrane peptidase-like |
| rs799623 | 7 | 110296372 | 0.6045667 | 0.5225333 | IMP2 inner mitochondrial membrane peptidase-like |
| rs9886295 | 7 | 110272524 | -0.61787 | -0.524 | IMP2 inner mitochondrial membrane peptidase-like |
| rs10225614 | 7 | 110390663 | -0.63203 | -0.5307 | IMP3 inner mitochondrial membrane peptidase-like |
| rs740341 | 7 | 110284409 | -0.59237 | -0.5254 | IMP4 inner mitochondrial membrane peptidase-like |
| rs799616 | 7 | 110308139 | -0.64623 | -0.5135 | IMP5 inner mitochondrial membrane peptidase-like |
| rs6917532 | 6 | 129830351 | -0.5446 | -0.51733 | laminin, alpha 2 |
| rs7741996 | 6 | 129838313 | -0.67107 | -0.57827 | laminin, alpha 2 |
| rs495418 | 1 | 181426287 | -0.51687 | -0.51887 | laminin, gamma 2 |
| **rs1539177** | **13** | **46198056** | **0.5276667** | **0.5500667** | **leucine-rich repeats and calponin homology (CH) domain containing 1** |
| **rs17068697** | **13** | **46191816** | **0.5793667** | **0.5600667** | **leucine-rich repeats and calponin homology (CH) domain containing 1** |
| **rs2236592** | **13** | **46193917** | **-0.55503** | **-0.55213** | **leucine-rich repeats and calponin homology (CH) domain containing 1** |
| **rs9534475** | **13** | **46190612** | **-0.55447** | **-0.57853** | **leucine-rich repeats and calponin homology (CH) domain containing 1** |
| rs10183908 | 2 | 142248888 | 0.656867 | 0.581167 | low density lipoprotein receptor-related protein 1B |
| rs352985 | 2 | 142310153 | 0.6666 | 0.578467 | low density lipoprotein receptor-related protein 1B |
| rs7558916 | 2 | 142250189 | 0.686033 | 0.6186 | low density lipoprotein receptor-related protein 1B |
| rs10496896 | 2 | 142253289 | -0.87483 | -0.50043 | low density lipoprotein receptor-related protein 1B |
| rs1541976 | 2 | 142319382 | -0.8425 | -0.54923 | low density lipoprotein receptor-related protein 1B |
| rs1052030 | 11 | 76531431 | 0.5834667 | 0.5712 | myosin VIIA |
| rs4945149 | 11 | 76529687 | 0.6121333 | 0.5861333 | myosin VIIA |
| rs10083466 | 14 | 79054471 | 0.5926333 | 0.5869333 | neurexin 3 |
| rs11845632 | 14 | 79023567 | 0.5457333 | 0.5682667 | neurexin 3 |
| rs2196447 | 14 | 79020241 | 0.5855667 | 0.5635333 | neurexin 3 |
| rs9364635 | 6 | 162362566 | 0.5084333 | 0.5181 | Parkinson disease (autosomal recessive, juvenile) 2, |
| rs1624390 | 6 | 162296972 | 0.5992 | 0.5569 | Parkinson disease (autosomal recessive, juvenile) 2, parkin |
| rs1784594 | 6 | 162302724 | 0.5606 | 0.5402667 | Parkinson disease (autosomal recessive, juvenile) 2, parkin |
| rs1784597 | 6 | 162295899 | 0.5815667 | 0.5669667 | Parkinson disease (autosomal recessive, juvenile) 2, parkin |
| rs1893895 | 6 | 162360677 | 0.5265333 | 0.5154333 | Parkinson disease (autosomal recessive, juvenile) 2, parkin |
| rs1342362 | 1 | 240618321 | 0.5574 | 0.5445667 | phospholipase D family, member 5 |
| rs2580222 | 1 | 240625569 | 0.5653333 | 0.5503333 | phospholipase D family, member 5 |
| rs2654861 | 1 | 240620287 | 0.5414667 | 0.5872667 | phospholipase D family, member 5 |
| rs10494991 | 1 | 213287222 | -0.5077 | -0.52267 | potassium channel, subfamily K, member 2 |
| rs11120481 | 1 | 213307034 | -0.52103 | -0.51523 | potassium channel, subfamily K, member 2 |
| rs11191686 | 10 | 105177736 | -0.53433 | -0.5176 | programmed cell death 11 |
| rs3740382 | 10 | 105169204 | -0.57367 | -0.51827 | programmed cell death 11 |
| rs11154102 | 6 | 123009696 | -0.54867 | -0.58827 | protein kinase (cAMP-dependent, catalytic) inhibitor beta |
| rs17052989 | 6 | 123022258 | -0.61467 | -0.59833 | protein kinase (cAMP-dependent, catalytic) inhibitor beta |
| rs6569264 | 6 | 123025942 | -0.62367 | -0.61037 | protein kinase (cAMP-dependent, catalytic) inhibitor beta |
| rs9388105 | 6 | 122986839 | -0.5701 | -0.58273 | protein kinase (cAMP-dependent, catalytic) inhibitor beta |
| rs208807 | 20 | 36932553 | -0.6057 | -0.60617 | protein phosphatase 1, regulatory (inhibitor) subunit 16B |
| rs6065094 | 20 | 36886608 | -0.61583 | -0.58647 | protein phosphatase 1, regulatory (inhibitor) subunit 16B |
| rs1721175 | 3 | 77733384 | -0.519 | -0.5238 | roundabout, axon guidance receptor, homolog 2 (Drosophila |
| rs796527 | 3 | 77702083 | -0.57057 | -0.55843 | roundabout, axon guidance receptor, homolog 2 (Drosophila |
| rs1165160 | 6 | 25972435 | -0.87223 | -0.50007 | solute carrier family 17 (sodium phosphate), member 3 |
| rs1141034 | 6 | 25888311 | 0.5377667 | 0.5194 | solute carrier family 17 (sodium phosphate), member 4 |
| rs4712970 | 6 | 25878686 | 0.6083667 | 0.5629333 | solute carrier family 17 (sodium phosphate), member 4 |
| rs1892248 | 6 | 25876893 | 0.617433 | 0.570267 | solute carrier family 17 (sodium phosphate), member 4 |
| rs3778272 | 6 | 25875640 | 0.650367 | 0.571067 | solute carrier family 17 (sodium phosphate), member 4 |
| rs1317510 | 6 | 25886903 | -0.60187 | -0.56843 | solute carrier family 17 (sodium phosphate), member 4 |
| rs9358884 | 6 | 25864571 | -0.61687 | -0.5649 | solute carrier family 17 (sodium phosphate), member 4 |
| rs264653 | 2 | 159635095 | 0.5339333 | 0.5439 | tetratricopeptide repeat, ankyrin repeat and coiled-coil containing 1 |
| rs447106 | 2 | 159608524 | 0.5757667 | 0.6067 | tetratricopeptide repeat, ankyrin repeat and coiled-coil containing 1 |
| rs2839517 | 21 | 42738366 | -0.60533 | -0.554 | ubiquitin associated and SH3 domain containing A |
| rs884340 | 21 | 42739607 | -0.51757 | -0.51307 | ubiquitin associated and SH3 domain containing A |
| rs10114937 | 9 | 35261817 | -0.58527 | -0.55767 | unc-13 homolog B (C. elegans) |
| rs10758303 | 9 | 35279544 | -0.60193 | -0.61867 | unc-13 homolog B (C. elegans) |
| rs589611 | 15 | 52606857 | 0.870367 | 0.501167 | unc-13 homolog C (C. elegans) |
| rs8039506 | 15 | 52269214 | 0.639933 | 0.5485 | unc-13 homolog C (C. elegans) |
| rs12471649 | 2 | 28996929 | 0.5506333 | 0.5164667 | WD repeat domain 43 |
| rs4666141 | 2 | 29017526 | 0.5581 | 0.5197333 | WD repeat domain 43 |
| rs3790088 | 16 | 68435662 | 0.6079667 | 0.5652333 | WW domain containing E3 ubiquitin protein ligase 2 |
| rs4247109 | 16 | 68360944 | 0.6109 | 0.6116 | WW domain containing E3 ubiquitin protein ligase 2 |
| rs2088747 | 16 | 68425844 | -0.5993 | -0.56823 | WW domain containing E3 ubiquitin protein ligase 2 |
| rs16823443 | 3 | 116194043 | 0.5615 | 0.5332 | zinc finger and BTB domain containing 20 |
| rs1506028 | 3 | 116176944 | 0.614233 | 0.603633 | zinc finger and BTB domain containing 20 |
| rs12639377 | 3 | 116319386 | -0.70643 | -0.58687 | zinc finger and BTB domain containing 20 |
| rs250313 | 19 | 8786400 | 0.5804333 | 0.5291333 | zinc finger protein 558 |
| rs250316 | 19 | 8782033 | 0.5826333 | 0.515 | zinc finger protein 558 |

The bold part represents four SNPs in LRCH1 gene.
